# Improvement in toxicity in high risk prostate cancer patients treated with image-guided intensity-modulated radiotherapy compared to 3D conformal radiotherapy without daily image guidance

**DOI:** 10.1186/1748-717X-9-44

**Published:** 2014-02-04

**Authors:** Joen Sveistrup, Per Munck af Rosenschöld, Joseph O Deasy, Jung Hun Oh, Tobias Pommer, Peter Meidahl Petersen, Svend Aage Engelholm

**Affiliations:** 1 Department of Radiation Oncology, Section 3994, Rigshospitalet, Blegdamsvej 9, Copenhagen 2100, Denmark; 2 Department of Medical Physics, Memorial Sloan-Kettering Cancer Center, New York, New York, USA; 3 Department of Oncology, Rigshospitalet, Copenhagen, Denmark

**Keywords:** Prostate cancer, Radiotherapy, Image-guided radiotherapy (IGRT), Intensity-modulated radiotherapy (IMRT), Toxicity

## Abstract

**Background:**

Image-guided radiotherapy (IGRT) facilitates the delivery of a very precise radiation dose. In this study we compare the toxicity and biochemical progression-free survival between patients treated with daily image-guided intensity-modulated radiotherapy (IG-IMRT) and 3D conformal radiotherapy (3DCRT) without daily image guidance for high risk prostate cancer (PCa).

**Methods:**

A total of 503 high risk PCa patients treated with radiotherapy (RT) and endocrine treatment between 2000 and 2010 were retrospectively reviewed. 115 patients were treated with 3DCRT, and 388 patients were treated with IG-IMRT. 3DCRT patients were treated to 76 Gy and without daily image guidance and with 1–2 cm PTV margins. IG-IMRT patients were treated to 78 Gy based on daily image guidance of fiducial markers, and the PTV margins were 5–7 mm. Furthermore, the dose-volume constraints to both the rectum and bladder were changed with the introduction of IG-IMRT.

**Results:**

The 2-year actuarial likelihood of developing grade > = 2 GI toxicity following RT was 57.3% in 3DCRT patients and 5.8% in IG-IMRT patients (p < 0.001). For GU toxicity the numbers were 41.8% and 29.7%, respectively (p = 0.011). On multivariate analysis, 3DCRT was associated with a significantly increased risk of developing grade > = 2 GI toxicity compared to IG-IMRT (p < 0.001, HR = 11.59 [CI: 6.67-20.14]). 3DCRT was also associated with an increased risk of developing GU toxicity compared to IG-IMRT.

The 3-year actuarial biochemical progression-free survival probability was 86.0% for 3DCRT and 90.3% for IG-IMRT (p = 0.386). On multivariate analysis there was no difference in biochemical progression-free survival between 3DCRT and IG-IMRT.

**Conclusion:**

The difference in toxicity can be attributed to the combination of the IMRT technique with reduced dose to organs-at-risk, daily image guidance and margin reduction.

## Background

Radiotherapy in combination with androgen-deprivation therapy (ADT) is a well-documented treatment option for high risk prostate cancer (PCa) [1]. With intensity modulated radiotherapy (IMRT) it is possible to deliver high doses to the target and at the same time reduce the dose to the surrounding normal tissue [2]. Recently, the concept of image guided radiotherapy (IGRT) has been introduced in the treatment of PCa. IGRT is often based on the implantation of fiducial gold markers in the prostate. With markers implanted in the prostate, the position of the prostate can be verified before each treatment fraction using portal imaging. This limits the interfractional variability in the position of the prostate and as a consequence the PTV margins can be reduced [3]. In theory, a reduction in PTV margins should reduce the dose to the organs at risk and consequently result in less toxicity [4]. However, there is concern that large margin reductions might increase the risk of geographical miss of the target, which might result in higher rates of local failure [5].

In our centre, the RT for PCa has changed during the past decade. In 2000–2005 patients were treated with 3D conformal radiotherapy (3DCRT) to 76 Gy and without daily image guidance. In 2005, we introduced daily image-guided intensity modulated radiotherapy (IG-IMRT) based on implanted markers in the prostate. With the introduction of IG-IMRT, the PTV margins were reduced substantially, and the dose was increased to 78 Gy.

The purpose of this study is to compare toxicity and biochemical progression-free survival between patients treated with IG-IMRT and 3DCRT for high risk PCa. We hypothesize that IG-IMRT and reduced margins result in less toxicity compared to 3DCRT with broader margins and without daily image guidance. On the other hand, given the accuracy of IGRT the reduction in margins should not compromise the level of tumor control in IG-IMRT patients.

## Methods

### Patients

A total of 503 high risk PCa [6] patients treated with radiotherapy (RT) in the Department of Radiation Oncology at Rigshospitalet, Copenhagen, Denmark between January 1st 2000 and December 31 2010 were retrospectively reviewed. The definition of high risk disease was based on the d’Amico classification and was characterized by a T-stage ≥ T2c or Gleason Score ≥8 or Prostate Specific Antigene (PSA) level >20 ng/ml.

Between 2000–2005, 115 patients were treated with 3DCRT to 76 Gy without daily image guidance. Between 2005–2010, 388 patients were treated with IG-IMRT to 78 Gy. The large difference in the number of patients in the two cohorts can be explained largely by increased referral due to both increasing incidence of PCa as well as higher acceptance of RT as a relevant therapy for PCa. Pelvic node irradiation was not used in any of the patients.

The diagnosis of PCa was based on either transrectal ultrasound (TRUS) with biopsies or transurethral resection of the prostate (TUR-P). Histopathological samples were centrally reviewed. Patients were staged according to the American Joint Committee on Cancer staging system. There were no signs of lymph node metastases or distant metastases. Patient characteristics are shown in Table 1.

**Table 1:** Patient characteristics

**Categorical characteristic**	**3DCRT *n = 115***	**IG-IMRT *n = 388***	**P value**
	**No patients (%)**	**No patients (%)**	
Performance status			0.180
0	110 (96)	367 (95)	
1	5 (4)	21 (5)	
Comorbidity*			0.070
Yes	66 (60)	215 (56)	
No	44 (40)	166 (44)	
Smoking#			0.091
Yes	30 (32)	106 (29)	
No	65 (68)	255 (71)	
T-stage			0.190
≤T2a	19 (17)	96 (25)	
T2b	3 (2)	10 (2)	
≥T2c	92 (81)	281 (73)	
Gleason score			<0.001
5-6	42 (44)	49 (13)	
7	21 (22)	197 (51)	
8-10	33 (34)	141 (36)	
Adjuvant ADT			0.019
Yes	72 (88)	350 (95)	
No	10 (12)	20 (5)	
Type of ADT			0.054
GnRH agonist	100 (89)	365 (95)	
Antiandrogen	12 (11)	21 (5)	
**Continuous characteristic**	**Median (range)**	**Median (range)**	**P value**
Age (y)	63 (52–75)	66 (49–76)	<0.001
PSA (ng/ml)	32.7 (4.2-150)	24.9 (4.2-200)	<0.001
PPB† (%)	75 (17–100)	67 (5–100)	0.006
Follow-up (yr)	8.2 (0.1-12.5)	3.5 (0.3-7.1)	<0.001

*Any comorbidity at the time of diagnosis.

#Smoking at the time of diagnosis.

†Percentage of positive (malignant) biopsies.

Comorbidity was present in 281 patients (56%). The five most common comorbidities were hypertension (95 patients), ischemic heart disease (32 patients), obesity (35 patients), diabetes (28 patients) and chronic obstructive pulmonary disease or asthma (28 patients). The majority of patients with comorbidity had more than one comorbidity.

Androgen deprivation therapy (ADT) guidelines were the same for the entire cohort. The ADT consisted of 3 months of neo-adjuvant treatment followed by two years of adjuvant treatment with primarily a GnRH (Gonadotropin releasing hormone) agonist combined with an antiandrogen in 30 days to account for the flare reaction. Alternatively, an antiandrogen was used as monotherapy during the whole ADT. Only two patients (one in the 3DCRT and one in the IG-IMRT group) did not receive neo-adjuvant ADT. A total of 30 patients did not receive adjuvant ADT.

Follow-up evaluations were performed in the Departments of Urology at either 3 or 6 months intervals. Genitourinary (GU) and gastrointestinal (GI) toxicity were assessed retrospectively based on patient records and was reported at the end of the RT, and at 6, 12 and 24 months after RT. The grading of toxicity was based on the Common Terminology Criteria for Adverse Events (CTC version 4.0) and was performed by a single physician (JS). GI toxicity included diarrhea, rectal hemorrhage, rectal pain and rectal incontinence. GU toxicity included dysuria, pollakisuria, incontinence, bladder spasms, hematuria, urinary retention or non-specified symptoms. Erectile dysfunction was not reported in this study due to very low reporting in the patient records. Furthermore, the majority of patients received two years of adjuvant ADT.

Biochemical progression was based on the Phoenix definition, i.e. a rise in PSA by 2 ng/mL or more above the nadir PSA level [7]. In case salvage ADT was commenced due to PSA progression before relapse according to the Phoenix definition, the date of the start of salvage ADT was defined as the date of biochemical progression.

Approval from the National Ethics Committee was not needed since this was a retrospective study, which had no influence on the treatment of the patients. The study was approved by the Regional Data Protection Agency.

### Treatment

#### 3DCRT (years 2000–2005)

3DCRT was delivered using the BeamCath® technique, see [8] for full details. Briefly, the BeamCath is a special urethral catheter containing high-density fiducial markers and it was placed in the patient’s urethra and bladder. The markers can be visualized using electronic portal vision device (Varian Medical Systems, Palo Alto, US) and allows for better vizualisation of the urethral part of the prostate. The catheter was used during the treatment planning procedure and then only during the first 3 fractions. The planning target volume (PTV) for the first 3 fractions included the prostate with no margin using two opposing lateral photon beams of 18 MV, shielding the rectum. Thus, the BeamCath technique allows the dose to be escalated from 70 Gy to 76 Gy without an increase in toxicity [9].

The remaining treatment was delivered without the BeamCath using a standard 4-field box technique with weekly port film verification based on bony landmarks. The clinical target volume (CTV) was defined as the prostate visible on CT. The seminal vesicles (SM) were included in the target if the risk of involvement exceeded 10% according to Partin’s Nomogram [10], or if SM biopsies showed malignant involvement. Fourth and fifth fractions were delivered with a margin to the PTV of 1 cm and the remaining 33 fractions with a margin of 2 cm. Treatment plans were made based on ICRU (International Commission of Radiation Unit and Measurement) guidelines with a dose variation of 95 to 107% within the PTV. The prescription dose was 76 Gy (2 Gy per fraction, 5 fractions per Week).

Dose volume constraints were as follows: Less than 20% of the outlined rectal volume should receive doses >70 Gy. For the bladder, the mean dose should not exceed 80% of the dose to the ICRU reference point, i.e. 60.8 Gy.

#### IG-IMRT (years 2005–2010)

IMRT was delivered with a 5-beam sliding window technique (fixed-angle IMRT) in the years 2005–2008 and with a single beam rotational technique (VMAT - RapidArc*®,* Varian Medical Systems, Palo Alto, US) in the years 2008–2010; see [11] for full details. Fixed-angle IMRT was used in 236 patients and VMAT was used in 152 patients.

Before RT planning, three gold fiducial markers were placed in the prostate under ultrasound guidance. Patients were subsequently scanned using CT and MR (1.5 T and T1 and T2 weighted imaging) in the supine treatment position. MR and CT data sets were exported and manually matched using the gold markers as reference. Bladder, rectum, femoral heads and prostate were defined using MR/CT. The treatment position was verified daily before treatment based on the implanted markers using orthogonal stereoscopic kilovoltage x-ray images (ExacTrac®, BrainLab AG, Germany) and digitally reconstructed radiographs from the simulation CT.

The CTV was defined as the prostate and any extraprostatic tumor visible on MR. The two proximal cm of the SM were included in the CTV, if the risk of involvement exceeded 10% according to Partin’s Nomogram [10]. In case SM biopsies showed malignant involvement, the entire SM was included in the CTV. The PTV margin was 5 mm in the right-left and anterior-posterior planes and 7 mm in the superior-inferior plane. Image registrations and plans were made using the Eclipse treatment planning system (Varian Medical Systems, Palo Alto, CA, US) and based on ICRU guidelines. The prescription dose was 78 Gy (2 Gy per fraction, 5 fractions per week).

The dose-volume constraints were as follows: For the rectum, less than 60% of the volume received 40 Gy, less than 50% received 50 Gy and less than 10% of the volume received 70 Gy. For the bladder, less than 50% of the volume received 50 Gy.

### Statistical analysis

The patient characteristics in the two groups were compared using a Chi-square test or a Fischer’s exact test for categorical variables and an independent sample t-test or the Mann–Whitney test for continuous variables.

The Kaplan Meier method was used to calculate the 2-year actuarial probability of developing ≥2 grade GI and GU toxicity. The same test was used to calculate the 3-year actuarial biochemical progression-free survival probability.

A Cox regression analysis was used to identify predictors of developing grade ≥2 GI and GU toxicity and biochemical progression. The following covariates were applied in the Cox regression analyses of toxicity: Age, performance status (PS), comorbidity, smoking, treatment technique (3DCRT vs IG-IMRT), and the use of ADT. In the cox regression analysis of biochemical progression the T-stage, percentage of positive (malignant) biopsies (PPB), Gleason score and PSA level were furthermore included.

Age, PPB, Gleason score and PSA were treated as continuous variables and the remaining as categorical variables.

## Results

**Figure 1:**
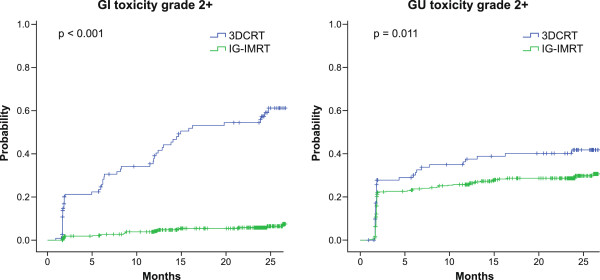
Comparison of the actuarial likelihood of developing a grade ≥2 GI and GU toxicity, respectively, during a 2-year follow-up between patients treated with 3DCRT and IG-IMRT.

The 2-year actuarial likelihood of developing grade ≥2 GI toxicity following RT was 57.3% for 3DCRT and 5.8% for IG-IMRT (p < 0.001). For grade ≥2 GU toxicity the corresponding numbers were 41.8% and 29.7% (p = 0.011), respectively (Figure 1).

Predictors of toxicity and biochemical progression are shown in Tables 2 and 3, respectively. For the continuous variables age, PPB, Gleason score and PSA, the results indicate the hazard ratio per one unit increase in the variables. For PS the results indicate the hazard ratio for patients with PS = 1 compared to patients with PS = 0. For the T-stage the results indicate the hazard ratio for patients with T-stage ≥ T2c compared to patients with T-stage ≤ T2b.

**Table 2:** Univariate and multivariate analyses of grade +2 GI and GU toxicity

**GI Toxicity**	**Univariate analysis**			**Multivariate analysis**		
**Parameter**	**Hz**	**95% CI**	**p-value**	**Hz**	**95% CI**	**p-value**
Age	0.93	0.89-0.97	<0.001*	0.99	0.93-1.04	0.584
Performance status	1.67	0.73-3.85	0.227	1.84	0.70-4.82	0.218
Comorbidity	1.18	0.75-1.86	0.482	0.88	0.62-1.77	0.878
Smoking	1.05	0.62-1.77	0.853	0.71	0.51-1.58	0.708
3DCRT vs IG-IMRT	12.81	7.84-20.92	<0.001*	11.59	6.67-20.14	<0.001*
Adjuvant ADT	0.31	0.16-0.60	0.001*	0.27	0.13-0.56	<0.001*
**GU toxicity**	**Univariate analysis**			**Multivariate analysis**		
**Parameter**	**Hz**	**95% CI**	**p-value**	**Hz**	**95% CI**	**p-value**
Age	1.00	0.97-1.04	0.869	1.01	0.97-1.05	0.476
Performance status	1.80	0.99-3.24	0.052	1.68	0.89-3.18	0.111
Comorbidity	1.50	1.07-2.11	0.018*	1.50	1.02-2.21	0.038*
Smoking	1.27	0.89-1.81	0.185	1.28	0.88-1.88	0.200
3DCRT vs IG-IMRT	1.58	1.10-2.26	0.013*	1.72	1.11-2.67	0.015*
Adjuvant ADT	0.60	0.34-1.06	0.078	0.57	0.30-1.06	0.075

*Statistically significant.

**Table 3:** Univariate and multivariate analyses of biochemical progression

	**Univariate analysis**			**Multivariate analysis**		
**Parameter**	**Hz**	**95% CI**	**p-value**	**Hz**	**95% CI**	**p-value**
Age	0.93	0.90-0.97	0.001*	0.93	0.88-0.99	0.017*
Performance status	0.57	0.14-2.32	0.432	0.40	0.05-2.93	0.371
Comorbidity	1.02	0.66-1.58	0.931	1.03	0.56-1.82	0.928
Smoking	1.15	0.71-1.88	0.574	1.31	0.73-2.35	0.361
T-stage (≥T2c vs ≤ T2b)	0.71	0.44-1-16	0.170	0.58	0.29-1.17	0.128
PPB#	1.02	1.01-1.03	0.001*	1.01	1.00-1.02	0.038*
Gleason score	1.29	1.05-1.58	0.014*	1.04	0.80-1.39	0.759
PSA	1.00	1.00-1.01	0.555	1.00	0.99-1.01	0.433
3DCRT vs IG-IMRT	1.24	0.77-2.00	0.386	0.78	0.37-1.57	0.505
Adjuvant ADT	0.59	0.28-1.24	0.163	0.89	0.31-2.56	0.830

*Statistically significant.

#Percentage positive biopsies.

In the multivariate analysis, 3DCRT was associated with a significantly increased risk of developing grade ≥2 GI toxicity compared to the IG-IMRT group (p < 0.001, HR = 11.59 [CI: 6.67-20.14]). 3DCRT was also associated with a significantly increased risk of developing grade ≥2 GU toxicity compared to IG-IMRT (p = 0.015, HR = 1.72 [CI:1.11-2.67]). Furthermore, patients with comorbidity had a higher risk of developing GU toxicity compared to patients without comorbidity (p = 0.038, HR = 1.50 [CI:1.02-2.21]). Finally, the use of ADT was protective of GI toxicity (p < 0.001, HR = 0.23 [CI: 0.10-0.50]).

**Figure 2:**
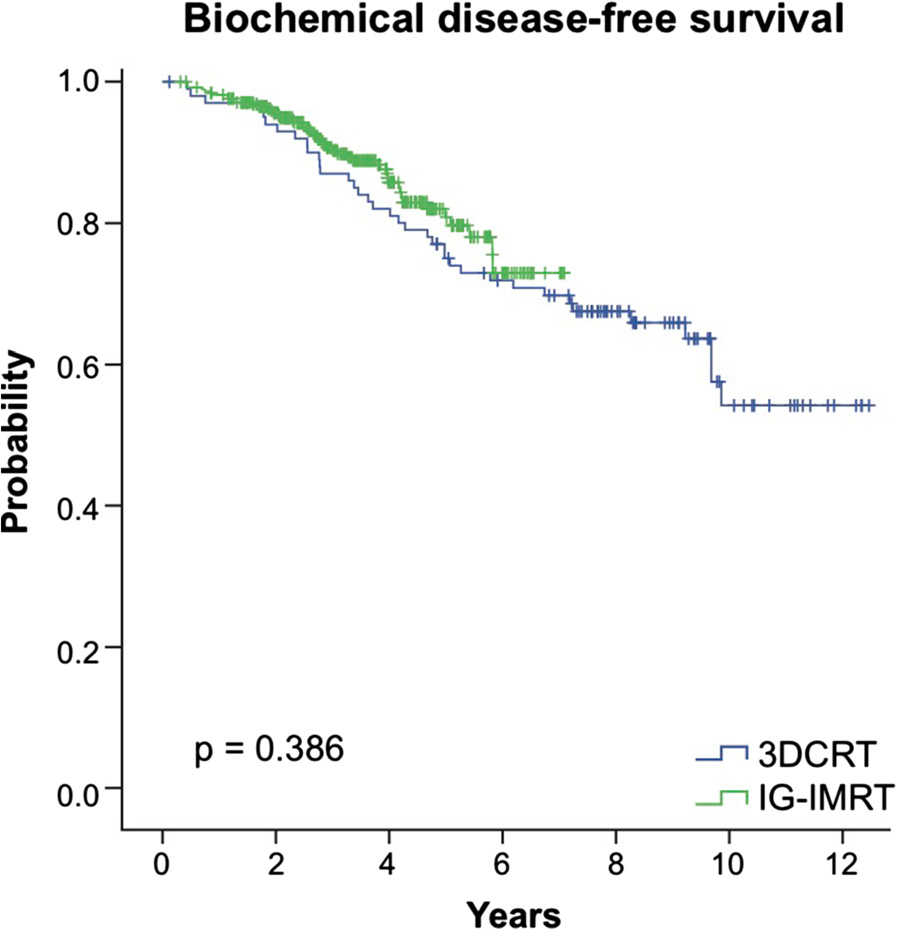
Comparison of the actuarial likelihood of developing biochemical relapse between patients treated with 3DCRT and IG-IMRT.

The 3-year actuarial biochemical progression-free survival probability was 86.0% for 3DCRT and 90.3% for IG-IMRT (p = 0.386) (Figure 2). As shown in the Cox regression analysis in Table 3, there was no significant difference between 3DCRT and IG-IMRT in the risk of developing biochemical progression (p = 0.505, HR = 0.78 [CI:0.37-1.57]). Increasing age was associated with a lower risk of developing biochemical progression (p = 0.017, HR = 0.93 [CI: 0.88-0.99]). Higher percentage of positive (malignant) biopsies (PPB) was predictive of biochemical progression (p = 0.038, HR = 1.01 [CI: 1.00-1.02]).

## Discussion

In this study, we compared toxicity and biochemical progression-free survival in high risk PCa patients treated with either of two RT techniques: 3DCRT without daily image guidance versus daily fiducial-based IG-IMRT. IG-IMRT was introduced in our centre in 2005 and was accompanied by a small dose escalation and a substantial reduction in PTV margins. To our knowledge, this is one of the largest studies to demonstrate the clinical effect of introducing IG-IMRT and smaller margins in the treatment of high risk PCa. In lack of randomized trials comparing RT techniques and different margins, it is important that we keep reporting clinical outcomes following the implementation of new radiation techniques [12].

**Figure 3:**
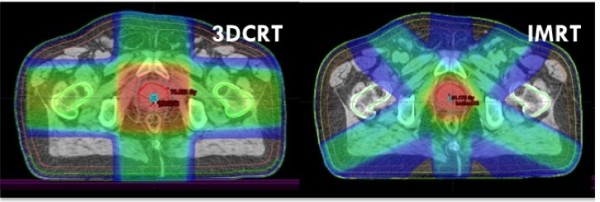
Left: Treatment plan for 3DCRT (2 cm PTV margin). Right: Treatment plan for fixed-angle IMRT (5–7 mm PTV margin).

We found that 3DCRT patients experienced significantly more toxicity than IG-IMRT patients. The observed difference in GI toxicity was large and can be attributed to the combination of the accuracy of IGRT, reduced PTV margins and IMRT-based dose sparing of the rectum [4,13]. Furthermore, the use of MR imaging in the treatment planning of IG-IMRT probably results in reduced dose delivery to the organs at risk due to smaller clinical target volumes [14]. We speculate that the major contributing factor for the large difference in GI toxicity is the large difference in PTV margins between the two groups. An example of a 3DCRT plan with 2 cm PTV margin and a fixed-angle IMRT plan with 5–7 mm PTV margins is shown in Figure 3. Assessment of the differences in dose-volume parameters between the treatment groups was beyond the scope of this clinical report.

Compared to other studies of non-image guided 3DCRT, we observed a relatively high degree of GI toxicity in our 3DCRT patients. However, our results are comparable to other reports of 3DCRT using the BeamCath technique [9,15]. Furthermore, the 2 cm PTV margin used in most fractions in 3DCRT is wider than what is commonly used in studies of 3DCRT without daily image guidance. The difference in GU toxicity between the two treatment groups was not nearly as significant as the difference in GI toxicity. A part of the explanation might be that the prostatic part of the urethra was not spared in either of the treatment groups [16]. Not surprisingly, patients with comorbidity seemed to have a higher risk of developing GU toxicity compared to patients without comorbidity.

Even though the entire cohort consisted of high risk patients, 30 patients did not receive adjuvant ADT. Interestingly, we found that the use of adjuvant ADT was independently associated with a lower risk of developing GI toxicity. Although short-term ADT has previously been shown to be protective of toxicity [17], the actual mechanism of such an effect remains unclear.

There was no significant difference in biochemical progression-free survival between the two treatment groups. The minor difference in prescribed dose was not expected to influence the biochemical progression-free survival to a large degree [18]. The major difference between the two groups besides the actual radiation technique was the use of daily image guidance and the size of the PTV margins. The 2 cm margin used in the majority of fractions with 3DCRT should be large enough to account for the interfractional and intrafractional motion of the prostate [19]. With the introduction of IG-IMRT, the margins were reduced to 5–7 mm due to higher accuracy of the dose delivery [20]. As mentioned in the introduction, concern has been raised that IGRT and a large reduction in PTV margins might lead to higher rates of local failure. Engels et al. [5] found that IGRT with 3–5 mm margins was associated with a poorer biochemical progression-free survival in patients treated with conformal arc therapy compared to non-IGRT and 6–10 mm margins. Our results are reassuring in that the substantial margin reduction from 2 cm to 5–7 mm did not seem to compromise the biochemical progression-free survival in IG-IMRT patients.

Both 3DCRT to 76 Gy and IG-IMRT to 78 Gy resulted in excellent tumor control. Our results are consistent with the results from other reports of high risk PCa [4,21]. The percentage of positive biopsies (PPB) was an independent predictor of biochemical progression. It would be easily understood if PPB were a more accurate marker of tumor advancement than the clinical T-stage, which is subject to a high degree of inter-observer variability. Increasing age was associated with a significantly lower risk of biochemical relapse, which might reflect that younger patients might have more aggressive tumors [22].

This study has several limitations. First of all, retrospective assessment of toxicity based on patient records involves a substantial degree of uncertainty. It is also a limitation that 13 of 41 of the relapses in the 3DCRT cohort were based on the initiation of salvage ADT due to PSA progression before relapse according to the Phoenix definition. This might falsely lower the biochemical progression-free survival in the 3DCRT group compared to the IG-IMRT group in which all relapses were based on the Phoenix definition. The two cohorts also differed significantly in baseline characteristics, which might affect the risk of biochemical progression differently in the two groups. Finally, as the median length of follow-up was relatively short in the IG-IMRT group, we were only able to report the 3-year biochemical progression-free survival rates. Thus, our results are somewhat premature, also considering that the large majority of patients received two years of adjuvant ADT.

## Conclusion

In this study, the combination of daily image guidance, IMRT and 5–7 mm PTV margins to 78 Gy resulted in significantly reduced GI and GU toxicity compared to 3DCRT to 76 Gy without daily image guidance and with 1–2 cm PTV margins. Importantly, the large reduction in margins did not seem to affect the level of biochemical tumor control in IG-IMRT patients. A major caveat is that this improvement may depend on the details of our implementation, and this should not be taken as a broad confirmation that any implementation of IG-IMRT with reduced margins is always an improvement over 3DCRT with larger margins.

## Competing interests

PMR received a grant from Varian Corp. JOD received a grant from Brainlab Corp. SAE received a grant from Brainlab Corp.

## Authors’ contributions

JS is responsible for the collection of all data, the design of the study and the writing of the manuscript. PMR and JOD have contributed to the analyses and interpretation of the results and have critically revised the manuscript. JHO carried out the statistical analyses. TP made the images in this study. PMP and SAE are responsible for the design of the study and have also critically revised the manuscript. All authors read and approved the final manuscript.
